# Feasibility study into self-administered training at home using an arm and hand device with motivational gaming environment in chronic stroke

**DOI:** 10.1186/s12984-015-0080-y

**Published:** 2015-10-09

**Authors:** Sharon M. Nijenhuis, Gerdienke B. Prange, Farshid Amirabdollahian, Patrizio Sale, Francesco Infarinato, Nasrin Nasr, Gail Mountain, Hermie J. Hermens, Arno H. A. Stienen, Jaap H. Buurke, Johan S. Rietman

**Affiliations:** Roessingh Research and Development, Roessinghsbleekweg 33b, 7522 AH Enschede, The Netherlands; Department of Biomechanical Engineering, University of Twente, Enschede, The Netherlands; Adaptive Systems Research Group, School of Computer Science, University of Hertfordshire, Hatfield, UK; Department of NeuroRehabilitation, IRCCS San Raffaele Pisana, Rome, Italy; School of Health and Related Research, University of Sheffield, Sheffield, UK; Department of Biomedical Signals and Systems, University of Twente, Enschede, The Netherlands; Physical Therapy and Human Movement Sciences, Northwestern University, Chicago, IL USA; MIRA institute for Biomedical Technology and Technical Medicine, University of Twente, Enschede, The Netherlands

**Keywords:** Stroke, Dynamic orthotic device, Upper extremity, Telemedicine, Home training, Rehabilitation games

## Abstract

**Background:**

Assistive and robotic training devices are increasingly used for rehabilitation of the hemiparetic arm after stroke, although applications for the wrist and hand are trailing behind. Furthermore, applying a training device in domestic settings may enable an increased training dose of functional arm and hand training. The objective of this study was to assess the feasibility and potential clinical changes associated with a technology-supported arm and hand training system at home for patients with chronic stroke.

**Methods:**

A dynamic wrist and hand orthosis was combined with a remotely monitored user interface with motivational gaming environment for self-administered training at home. Twenty-four chronic stroke patients with impaired arm/hand function were recruited to use the training system at home for six weeks. Evaluation of feasibility involved training duration, usability and motivation. Clinical outcomes on arm/hand function, activity and participation were assessed before and after six weeks of training and at two-month follow-up.

**Results:**

Mean System Usability Scale score was 69 % (SD 17 %), mean Intrinsic Motivation Inventory score was 5.2 (SD 0.9) points, and mean training duration per week was 105 (SD 66) minutes. Median Fugl-Meyer score improved from 37 (IQR 30) pre-training to 41 (IQR 32) post-training and was sustained at two-month follow-up (40 (IQR 32)). The Stroke Impact Scale improved from 56.3 (SD 13.2) pre-training to 60.0 (SD 13.9) post-training, with a trend at follow-up (59.8 (SD 15.2)). No significant improvements were found on the Action Research Arm Test and Motor Activity Log.

**Conclusions:**

Remotely monitored post-stroke training at home applying gaming exercises while physically supporting the wrist and hand showed to be feasible: participants were able and motivated to use the training system independently at home. Usability shows potential, although several usability issues need further attention. Upper extremity function and quality of life improved after training, although dexterity did not. These findings indicate that home-based arm and hand training with physical support from a dynamic orthosis is a feasible tool to enable self-administered practice at home. Such an approach enables practice without dependence on therapist availability, allowing an increase in training dose with respect to treatment in supervised settings.

**Trial registration:**

This study has been registered at the Netherlands Trial Registry (NTR): NTR3669.

## Background

In the chronic phase after stroke, most people still have motor problems [1], leading to difficulties in performing activities of daily living (ADL). Good arm and hand motor function is essential to perform ADL independently. Therefore, restoration of arm and hand function is a major objective in stroke rehabilitation. Research into motor relearning and cortical reorganization after stroke has provided a neurophysiological basis for those aspects that are important to stimulate restoration of arm function [2, 3]: functional exercises, at high intensity and with active involvement of the patient within a motivating environment [4, 5].

New ways of providing healthcare services, such as teleconsultation and remote monitoring and treatment in the patient’s home, often referred to as telerehabilitation, is widely considered as having a bright future in the framework of an innovative rehabilitative approach [6]. The use of a rehabilitation service within the home allows the user to exercise independently, in an intensive, active and functional way, in a familiar environment while having continuous access to training tools. This gives the patient a sense of control and autonomy, which might also contribute to a better treatment outcome [7]. To enhance high training adherence, exercises should be provided in a motivating training environment, for instance via computerized gaming including feedback about performance [8]. Furthermore, a computerized telerehabilitation service enables remote monitoring of movements and remote offline (indirect) supervision by a therapist. This should reduce the need for one-to-one treatment time and home visits. This can help relieve the pressure on today’s healthcare system which is challenged by an ageing society and increasing long-term conditions such as stroke [9]. It may also enable prolonged rehabilitation for those who may not be able to access it due to resource and service restrictions within the healthcare system. However, telerehabilitation for stroke is still in its infancy. Two reviews confirmed that only limited numbers of telerehabilitation studies in the stroke domain are currently available [10, 11]. More research is required including utilization [10], effectiveness and satisfaction [11] of telerehabilitation services after stroke. Thirteen of the 16 studies within both reviews involved telerehabilitation programs with direct, online supervision, such as videoconsulting or telephone calls. However, this still relies on therapist availability. Interestingly, both reviews did not address the effective training duration as performed by participants during the interventions, which is an essential factor in rehabilitation since this is (in relation to intensity of training) a prerequisite for motor relearning. In addition, this is of particular interest for studies involving remote treatment and offline supervision.

Developments in technology-mediated rehabilitation have made it possible to use rehabilitation robotics to provide safe and intensive training to people with mild to severe motor impairments after neurologic injury [12, 13]. Such devices can provide high-intensity, repetitive, task-specific, interactive training of the impaired upper extremity, and have the potential to more accurately quantify therapy and monitor patients’ progress. Rehabilitation robotics has been shown to be effective for the hemiparetic arm [14–20]. However, many training studies using robotic devices focus on either the proximal or distal arm only [14, 17, 21]. To improve independent use of the upper extremity in daily life, it is important to include functional movements of both the proximal and distal arm and hand into post-stroke training [22, 23]. Two examples are The Activities of Daily Living Exercise Robot (ADLER) [24] and Gentle/G system [25] which have the ability to train both reaching and grasping movements. A next step would be to use such technology at home to support self-administered training, without requiring direct therapist involvement continuously. This requires a training device to be compact and easily transportable.

The current study aims to address the previously mentioned aspects: using a dynamic wrist and hand orthosis in a motivational gaming environment for home rehabilitation to enable and support a high dose of self-administered training, facilitating both proximal and distal arm and hand exercises. In addition, participants are challenged to exercise at their maximum capacity. One of the major issues for the success of telerehabilitation concerns the question whether patients not only accept the technology and profit from it, but also whether they can effectively use the system, and in what dose. This is especially relevant in this study, in which participants were exposed to independent training at home, without direct supervision, using a device which physically interacts with the arm and hand. Since the technology-supported telerehabilitation system for the arm and hand was developed specifically for this study, the evaluation fits best within the first two stages of telemedicine evaluation. The first stage focuses on the feasibility and usability of the technology used in an experimental design with a small number of subjects. In the second stage, potential working mechanisms will be explored in a small group of subjects [26]. Therefore, the objective of this study was to examine feasibility (user acceptance, effective use) and potential clinical changes (in arm and hand function) of a technology-supported arm and hand training system at home in chronic stroke.

## Methods

### Participants

Three clinical sites in Europe (rehabilitation center Het Roessingh, Enschede, the Netherlands; IRCCS San Raffaele Pisana, Rome, Italy; and the University of Sheffield, Sheffield, United Kingdom) were involved in participant recruitment. Participants were recruited from local rehabilitation centers and regional hospitals and through private physical therapy providers. Inclusion criteria were: (1) patients had to be between 6 months and 5 years after stroke; (2) between 18 and 80 years of age; (3) clinically diagnosed with partial central paresis of the arm or hand due to stroke, but with at least 15° active elbow flexion and active finger flexion of at least a quarter of the passive range of motion; (4) living at home with internet access; (5) having a carer who is co-resident or closely involved in their care; (6) able to understand and follow instructions; (7) no additional orthopedic, neurological, or rheumatologic disease of the upper extremity; (8) and absence of severe neglect or (uncorrected) visual impairments. All participants provided written informed consent before participation. The study protocol was approved by the local ethics committees in all three sites.

### Study design

This feasibility study has a longitudinal design. Participants trained at home for six weeks using the newly developed training system. Arm and hand function was evaluated one week before training (T01), within one week after training (T08), and at two-month follow-up after the end of training (T15). User acceptance (motivation and usability) was evaluated within one week after training (T08). The training dose was automatically stored by the system during the six weeks of training.

### Intervention

Participants trained at home using games while they were supervised remotely, indirectly, by a healthcare professional (HCP). This means that the participant and the HCP do not need to be online at the same time. The HCPs involved in this study were trained clinical researchers (human movement scientists), physical therapists or occupational therapists. All participants were told that they could train at the time of day they preferred. The general recommendation for training was about 30 minutes of exercise per day, six days per week. They were allowed to practice additionally if they wished to. Firstly, two professionals installed and initialized the training system at the participant’s home. This involved checking that the system was fully operational and providing instructions to the participant and a family carer regarding safe usage. Instructions for usage were shown within the patient user interface of the system, and participants were also provided with a hard copy user manual. This user manual also included a telephone number which participants could call in case of technical failures or other needs for assistance of the system. During the first week following installation, the HCP contacted each participant twice to ensure their competence with the training system.

**Fig. 1:**
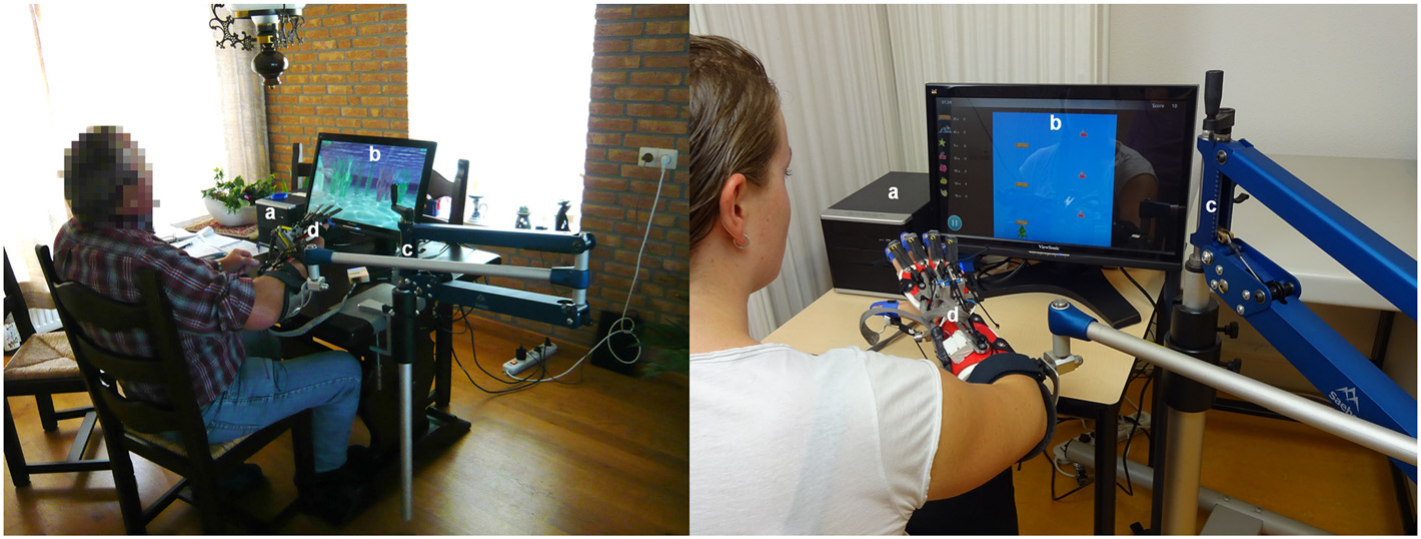
Technology-supported training system. **Left** = training system in use at participant’s home, **right** = more detailed overview of specific components, *a* Computer containing user interface and games, *b* Touchscreen showing one of the games, *c* SaeboMAS, *d* SCRIPT wrist and hand orthosis

The training system (Fig. 1) developed within the SCRIPT (Supervised Care and Rehabilitation Involving Personal Telerobotics) project [27] comprised: (A) a computer, (B) a touchscreen, (C) the Saebo Mobile Arm Support (SaeboMAS) (Saebo Inc., Charlotte NC, USA) and (D) the SCRIPT dynamic wrist and hand orthosis [28]. The SaeboMAS was used for gravity compensation of the proximal arm. The wrist and hand orthosis is a custom-designed exoskeleton which fits onto the forearm and hand. The mechanical design of the orthosis, in combination with the use of the SaeboMAS, allowed movements of the arm, wrist and hand within functional ranges during training. Trunk movements were not constrained. The orthosis interacted physically with the participant by providing extension forces to the wrist and fingers via passive leaf springs and elastic tension cords [28]. The amount of support could be adjusted to provide more or less of an offset force, to enable participants to train to their maximal capacity, with as little support as possible. For safety reasons, this amount of support was set by the HCP at the start of the training, based on a simple test to reach and grasp a soft ball several times. The amount of support was increased (decreased) when this test was perceived to be too difficult (easy), in addition to the participant’s opinion about comfort. The orthosis was equipped with sensors [28] to measure the range of motion of the wrist and fingers, and connected to the computer to allow game control and feedback on performance. The computer contained all the software components needed to complete a training session, including a user interface and games. The patient user interface, which was managed via a touchscreen, allowed the participant to select a game, play the game, and review his or her performance history (game scores and training duration). It also enabled the participant to contact the HCP by sending a message.

Three games with various categories were available for practice. The key gestures in the games were hand opening and closing (grasping), wrist flexion and extension, forearm pronation and supination, and elbow and shoulder movements (reaching). The integrated sensors in the orthosis enabled game control by active execution of gestures (for example wrist flexion or extension corresponded with an avatar diving under or jumping over obstacles on the screen) beyond a threshold of 80 % of the participant’s active range of motion. In this way, participants were challenged to exercise at their maximum capacity. This threshold was assessed prior to game playing through a calibration procedure of a few minutes, involving the gestures relevant for the game to be played. The main goals of the games were to provide a fun and engaging experience for interaction while assigning a task to the participant, and providing feedback about performance. In addition, visual (e.g. representation of scores during and after exercising) and auditory (e.g. applause and sound effects for correct movements) feedback was provided within the games to keep the participants motivated and engaged in training. The games consisted of various categories, which were classified in a schedule ranked according to increasing complexity. Games were considered more complex when they required multiple planes of movement (from 1D to 3D), when the number of gestures involved to control the game were increased, or when more difficult movements were needed (progression from proximal to distal movements, and gross to fine manipulation). This game difficulty schedule was used by the HCP weekly to provide the correct game categories to each participant. The HCP adjusted the training program remotely, by accessing the HCP user interface. The HCP visited this user interface daily, to check on potential messages from participants, and to follow the participants’ progress and training duration remotely. At that time, participants did not need to be online. If the HCP noticed no or low use, he or she sent a motivational message to the participant. The participant received this message the next time when he or she started a new training session. The decision to provide more complex categories of games was supported and verified by weekly home visits of the HCP to the participant’s home. During these weekly home visits participants performed a training session with the HCP of about 15 minutes of effective training. Above all, these visits were scheduled to answer potential questions, informally monitor progress, adjust the amount of support from the orthosis when needed, and encourage participants to practice more when training duration so far was low.

### Evaluation

#### User acceptance

The frequency and duration of practice (effective use and excluding calibration or system setup procedures) were recorded automatically by the system and displayed in the user interface. Motivation and usability of the system during training as experienced by the participants was measured using the Intrinsic Motivation Inventory (IMI) and the System Usability Scale (SUS), respectively. The IMI is a questionnaire with several dimensions that provides qualitative information about the content and level of motivation that a participant experiences during an intervention [29, 30]. It is scored on a seven-point Likert scale ranging from ‘not at all true’ to ‘very true’. A neutral score on the IMI is four, and a higher score means a more positive result on motivation.

The SUS is a 10-item scale giving a global view of subjective experience of usability. Questions are scored on a five-point Likert scale ranging from ‘strongly agree’ to ‘strongly disagree’. Scores are translated to 0–100 %, with a higher score meaning better usability [31]. Interventions that score in the 90s are exceptional, scores in the 70s and 80s are promising, and with SUS scores below 50 one can be almost certain that the product or intervention will have usability difficulties in the field [32, 33]. Both the IMI and SUS were completed during the post-intervention evaluation measurement (T08).

#### Arm and hand function

Clinical tests were used to quantify general arm function, before and after training and at two-month follow-up. The scales used are valid, standardized assessments, which were applied according to their specific protocols. The Action Research Arm Test (ARAT) evaluates coordination, dexterity and upper extremity function on four subtests (grasp, grip, pinch, and gross arm movement). The maximum score is 57 points [34–36]. The upper extremity part of the Brunnstrom Fugl-Meyer assessment (FM) evaluates motor status and the degree of synergy-development in the upper extremity, with a maximum score of 66 [37, 38]. Separate scores were also calculated for proximal (maximum = 42 points) and distal components of the FM (maximum = 24 points). The Motor Activity Log (MAL) is a semi-structured interview specifically designed for hemiparetic stroke patients. It assesses the perceived use of the paretic arm and hand (amount of use and quality of movement) during activities of daily living [39]. The maximum score for both subsections is five points. The Stroke Impact Scale (SIS) is a questionnaire which assesses eight domains related to function, activities, and participation. Each domain score has a range of zero to hundred percent [40, 41]. A higher score indicates better quality of life after stroke.

### Statistical analyses

Statistical analyses were performed using IBM SPSS Statistics 19 for Windows. All outcomes were inspected for normal distribution using histogram plots including normal curves and normal probability plots, and Shapiro-Wilk tests, prior to selection of appropriate statistical tests. Descriptive statistics (mean with standard deviation (SD) for normal distributed outcomes, or median with interquartile range (IQR) for non-parametric outcomes) were used to describe the participant characteristics and all outcome measures. Clinical variables over time were analyzed using mixed models repeated measures analysis with adjustments for multiple comparisons (Sidak), or the non-parametric equivalent, Friedman’s ANOVA. In case of significance for non-parametric variables, additional Wilcoxon Signed Ranks Test for multiple comparisons were performed, using adjusted *P*-values in accordance with the Holm-Bonferroni correction. Additionally, the relationship between training duration and training-induced changes was assessed with Pearson’s (or non-parametric Spearman’s) correlation coefficient. The level for significance was set at α < 0.05 for all statistical tests. When using the Holm-Bonferroni correction, significance levels corresponded with α < 0.0167, 0.025 and 0.05 (sorted in order of smallest to largest *P*-value, respectively).

## Results

### Participants

Twenty-four participants were included in the study. Three participants were lost during the study because of shoulder pain due to external causes, technical problems with the system, and a desire to not continue. No post-training data is available from these participants, and they were not included in data analysis. The characteristics of the remaining 21 participants are displayed in Table 1. The group involved 9.5 % mildly, 57.2 % moderately and 33.3 % severely affected stroke patients, based on a categorization of baseline FM score [42]. Data on the SIS are incomplete for two participants, so analysis for this outcome was performed over 19 participants.

**Table 1:** Participant characteristics at baseline

	Participants (*N* = 21)
Sex (male/female)^a^	10/11
Age (years)^b^	59 ± 13 (34–80)
Time post stroke (months)^b^	19 ± 14 (6–50)
Type of stroke (infarction/hemorrhage)^a^	19/2
Affected body side (right/left)^a^	7/14
Dominant arm (right/left)^a^	19/2
FM score (maximal 66 points)^b^	33.1 ± 15.8 (9–56)
ARAT score (maximal 57 points)^b^	25.7 ± 21.0 (3–55)

*Abbreviations: FM score* Fugl-Meyer assessment score at baseline, *ARAT score* Action Research Arm Test score at baseline

^a^Absolute numbers

^b^Mean ± standard deviation (range)

### User acceptance

**Fig. 2:**
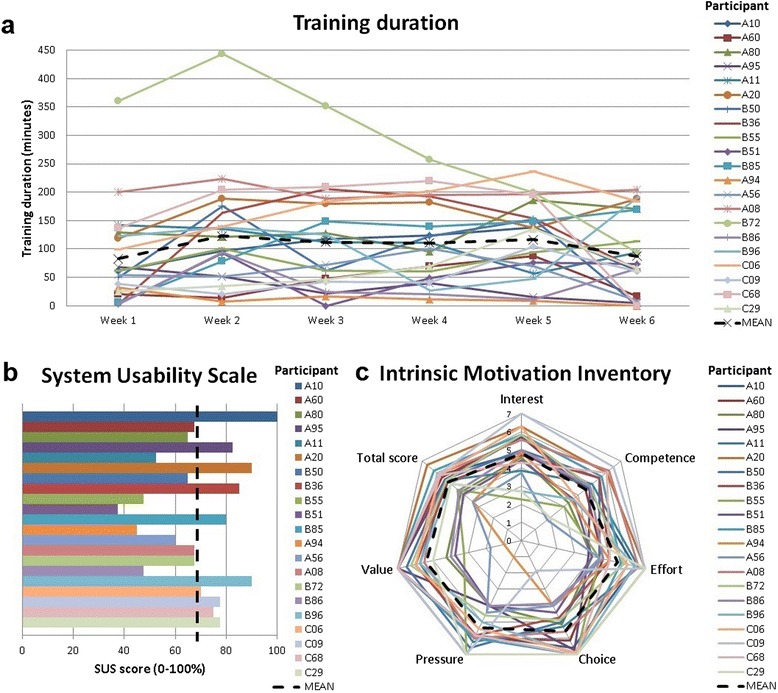
Individual results (colored lines) with group averages (dotted line) on user acceptance for *a* Training duration per week, *c* System Usability Scale and *c* Intrinsic Motivation Inventory

Figure 2 shows the main effects concerning user experience. Twenty-one participants used the system for six weeks, but with a large amount of variation in effective use between and within individuals. Mean training duration for the group, averaged per week over six weeks, was 105 minutes (SD = 66 minutes), whereas training duration ranged from 13 to 284 minutes per week across participants. In general, the motivation during training was positive, as reflected in the mean score on the IMI of 5.2 points (SD = 0.9 points). The mean score on the SUS is 69 % (SD = 17 %). On individual level, ten participants rated usability over 70 %, seven between 50 and 70 % and four below 50 %.

### Arm and hand function

The FM and SIS improved significantly after training (*P* = 0.002 and 0.004, respectively). Subsequent multiple comparison analysis showed significant improvements between T01 and T08, with a median improvement of 4 points for FM, and mean improvement of 3.7 points for SIS (Table 2). For FM, this was sustained at two-month follow-up with a median improvement of 3 points from T01 to T15, and showed a trend for SIS (mean improvement of 3.5 points), indicating improved quality of life after stroke. Considering specific SIS domains, the participants improved significantly on the domains strength (*P* = 0.004) and mobility (*P* = 0.014), and showed a trend for ADL (*P* = 0.077) after training. The proximal component of the FM showed a significant improvement over the training period (*P* = 0.012), with a mean improvement of 2.8 points between T01 and T08. This was sustained at two-month follow-up (3.1 points improvement overall). A trend was found for the distal component of the FM (*P* = 0.078). The other clinical outcomes showed no statistically significant changes after the six weeks of training or at follow-up (Table 2).

**Table 2:** Clinical outcome scores pre-training (T01), post-training (T08) and at follow-up (T15)

Outcome measures	(P-values)	T01	T08	T15	ΔT08-T01	ΔT15-T01	ΔT15-T08
FM^b^		37 (30)	41 (32)	40 (32)	+4 (6)	+3.0 (9)	+0 (4)
	(P = **0.002)**				**0.001**	**0.003**	0.312
FM_prox^a^		21.7 (8.2)	24.5 (8.8)	24.8 (9.0)	+2.8 (3.9)	+3.1 (4.4)	+0.3 (1.5)
	(P = **0.012)**				**0.010**	**0.012**	0.784
FM_dist^b^		15 (17)	16 (17)	16 (18)	+1 (2)	+1 (4)	+0 (1)
	(P = 0.078)						
ARAT^b^		20 (44)	29 (45)	29 (45)	+1 (2)	+1 (4)	+0 (3)
	(P = 0.101)						
MAL_AOU^b^		0.7 (2.0)	0.6 (2.2)	1.5 (1.9)	+0.0 (0.5)	+0.0 (0.8)	+0.0 (0.4)
	(P = 0.753)						
MAL_QOM^b^		0.9 (1.8)	1.1 (1.8)	1.5 (1.8)	+0.0 (0.6)	+0.2 (0.5)	+0.1 (0.4)
	(P = 0.229)						
SIS^a^		56.3 (13.2)	60.0 (13.9)	59.8 (15.2)	3.7 (4.9)	3.5 (6.6)	-0.1 (4.1)
	(P = **0.004**)				**0.003**	0.055	0.999
SIS_Strength^a^		44.7 (20.4)	55.4 (21.7)	49.4 (22.0)	+10.6 (11.8)	+4.7 (13.6)	-5.9 (15.0)
	(P = **0.004**)				**0.003**	0.352	0.169
SIS_Memory^b,c^		90.6 (46.9)	87.5 (31.3)	87.5 (34.4)	+0.0 (3.1)	+0.0 (6.3)	+0.0 (6.3)
	(P = 0.303)						
SIS_Emotion^a^		65.7 (15.9)	67.6 (15.9)	70.0 (16.3)	+1.9 (11.0)	+4.4 (12.0)	+2.5 (11.1)
	(P = 0.248)						
SIS_Communication^b,c^		85.7 (17.9)	89.3 (14.3)	85.7 (14.3)	+0.0 (14.3)	+0.0 (14.3)	+0.0 (10.7
	(P = 0.523)						
SIS_ADL^a^		57.1 (18.9)	59.3 (20.6)	54.6 (19.8)	+2.2 (7.4)	-2.5 (9.2)	-4.7 (9.9)
	(P = 0.077)						
SIS_Mobility^b,c^		55.0 (40.0)	67.5 (42.5)	70.0 (40.0)	+2.5 (12.5)	+5.0 (15.6)	+0.0 (15.0)
	(P = **0.014**)				0.051	0.029	0.955
SIS_Hand_function^b,c^		5.0 (55.0)	15.0 (60.0)	20.0 (55.0)	+0.0 (15.0)	+0.0 (15.0)	+0.0 (10.0)
	(P = 0.927)						
SIS_Participation^a^		48.9 (20.8)	50.9 (21.1)	56.0 (23.3)	+2.1 (16.0)	+7.2 (17.0)	+5.1 (13.9)
	(P = 0.132)						
SIS_Recovery^a^		52.3 (17.7)	54.0 (20.0)	53.0 (24.8)	+1.7 (11.3)	+0.8 (17.7)	+1.0 (14.8)
	(P = 0.813)						

Group mean or median values at T01, T08 and T15 are displayed, in addition to change scores over training (ΔT08-T01), after the end of training (ΔT15-T08) and over training and follow-up in total (ΔT15-T01). *P*-values of the main effect are displayed in the second column and in case of significance, *P*-values of post-hoc tests are displayed in the last three columns (significant *P*-values in **bold**).

***Abbreviations*** *: FM* Fugl-Meyer, *FM_prox* Fugl-Meyer proximal part, *FM_dist* Fugl-Meyer distal part, *ARAT* Action Research Arm Test, *MAL_AOU* Motor Activity Log Amount of Use, *MAL_QOM* Motor Activity Log Quality of Movement, *SIS* Stroke Impact Scale, *SIS_ADL* Stroke Impact Scale Activities of Daily Living section, *T01* baseline measurement pre-training, *T08* evaluation measurement post-training, *T15* two-month follow-up evaluation measurement

^a^Normally distributed variables displayed by mean (standard deviation) and analyzed by mixed models repeated measures analysis

^b^Non-parametric variables displayed by median (interquartile range) and analyzed by Friedman ANOVA

^c^ *N* = 19 because of incomplete dataset

**Fig. 3:**
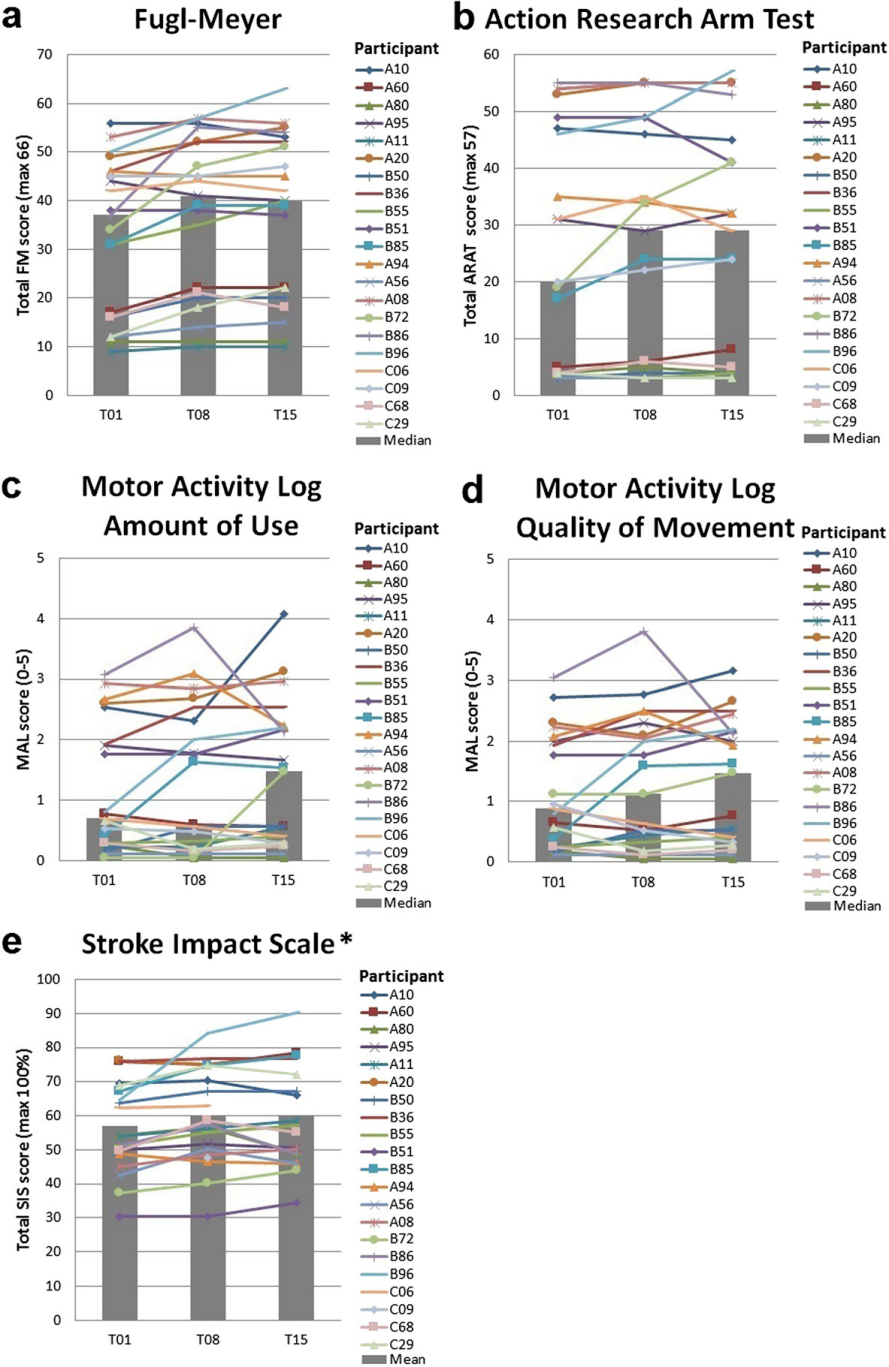
Individual (colored lines) and group (grey bars) results of the clinical scales for *a* Fugl-Meyer assessment, *b* Action Research Arm Test, *c* Motor Activity Log Amount of Use, *d* Motor Activity Log Quality of Movement and *E* Stroke Impact Scale. Abbreviations: FM = Fugl-Meyer, ARAT = Action Research Arm Test, SIS = Stroke Impact Scale, MAL = Motor Activity Log, T01 = baseline measurement pre-training, T08 = evaluation measurement post-training, T15 = two-month follow-up evaluation measurement. *Missing data Stroke Impact Scale: T01 *N* = 20, T08 *N* = 21, and T15 *N* = 19

Examination of the individual scores of each participant (Fig. 3) shows that some participants showed substantial improvements on clinical outcomes. Eight of the 21 participants reached minimal clinically important differences (MCID) for FM of 10 % improvement [43]. ARAT scores showed less marked improvements, with three participants reaching the MCID of 10 % improvement [43]. Seventeen of the 21 participants reported improvements in quality of movement of the affected arm in daily life on the MAL, of which 14 also reported improved amount of use. Of those, seven achieved MCID improvements of 0.5 points [44] for amount of use, and five for quality of movement on the MAL.

**Fig. 4:**
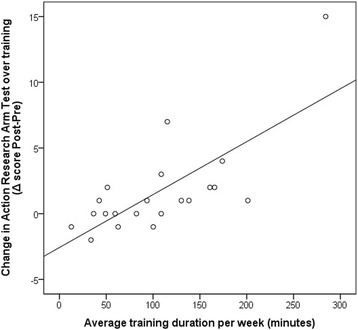
Scatter plot of average training duration and changes in Action Research Arm Test score over training

When examining the relationship between clinical outcome changes and training duration, a correlation was observed for dexterity. A moderate-strong, significant correlation was found between ARAT changes and training duration (Spearman’s rho = 0.686, *P* = 0.001) (Fig. 4), which means that a higher training duration is associated with a larger improvement in arm and hand dexterity.

## Discussion

The current study is one of the first studies in which technology-supported arm and hand training is performed in the patient’s home, where participants independently used a training device for both the proximal and distal arm and hand, which physically interacted with the participants, without direct supervision of an HCP. The objective was to examine feasibility (in terms of user acceptance and effective use) and potential clinical changes of the use of the training system. Results showed high marginal usability with potential for application in the field and motivation during training was good, which was reflected in a fair amount of effective use of 105 minutes per week. In addition, arm function improved over training and was sustained at two-month follow-up. Taking into account cost estimates as calculated within the SCRIPT project [27], home training with indirect supervision seems not only to have clinical value, but could also be economically viable in comparison with conventional rehabilitation and even home training as applied in many research studies nowadays. Within one year, this technology-supported training for six weeks per patient can be more cost-efficient when compared to dose-matched conventional therapy in the clinic with direct supervision by a therapist. Remotely monitored and physically supported arm and hand training at home showing feasibility and potential clinical value implies that an approach without direct, online therapist supervision should have the potential to allow practice without dependence on therapist availability. This might enable increased dose of training with respect to supervised treatment in clinical settings. The results obtained in our study correspond with those found in another home-based study, recently published by Sivan et al. [45]. They evaluated the feasibility of a robotic device for proximal arm training that can be used independently at home by stroke survivors with upper limb weakness. On group level, they found a mean training duration of 520 minutes during the 8-week study (which is about 65 minutes per week), and improvements on FM and ARAT of 1 point and 3 points, respectively. These improvements are comparable to the findings reported here, although these were slightly less pronounced on function level compared to the present study [45].

A review by Coupar et al. [46] involved four studies of in-home telerehabilitation for the upper limb after stroke. They found home-based upper limb programs to be no more or no less effective for arm motor impairment outcomes compared to upper limb programs conducted in hospitals [46]. In all included studies, the patients were (remotely) supervised at a fixed time, and without examining the effective amount of self-administered training at home. This is similar to other studies, in which a therapist in the clinic conducted treatment sessions with patients located at home [47], or in a different room in the clinic [48, 49]. In those studies the training sessions were scheduled beforehand, with about five hours (or more) practice per week. In our study, the HCP visited the participants only once per week (of which maximal 15 minutes of effective training), and patients were able to make their own decisions about their training schedule, without further direct real-time supervision of a therapist. The rationale for this was to remove the training constraints, and increase therapy availability. However, this makes it difficult to compare the effective amount of training in the above-mentioned studies with our study. The achieved training duration of approximately 105 minutes per week (about 15 minutes per day on average) suggests that stroke patients do have the incentive to train independently at home. When comparing specifically with recent studies into home-based self-administered upper limb therapy programs after stroke, the presently recorded effective use equals or even exceeds the adherence in those studies [45, 50, 51]. It is important to note that this 15 minutes of training stored in the portal involved only effective training time, which does not take into account for example donning and doffing of the orthosis and the calibration procedure. The total time participants spent on the self-administered training in this study would therefore be higher.

Usability of the system was rated sufficient, but the system might need improvements, which will be taken into account for further development of the system. When the technology has matured more, this should be tested in a large cohort study or cohort multiple randomized controlled trial as the next stage in telemedicine evaluation [26], to investigate clinical effects and explore if the results found here are comparable to conventional rehabilitation. Products or interventions with SUS scores below 70 % should be considered a candidate for increased scrutiny and continued improvement [33]. On individual level, half of the participants rated usability over 70 %, which means that the technology will have good to excellent chances for acceptance in the field, whereas only four participants scored SUS below 50 % (a product or intervention will probably have usability difficulties) [32, 33]. One of the examples of usability issues encountered by participants was unstable recording of the arm movements in space, as measured by an inertial sensor, which did not allow participants to fluently control and play one of the games involving reaching movement. Remarkably, two of the four participants with low scores on usability still performed a fair amount of training per week (>80 minutes). These two participants were not accustomed to new technologies and computers and found it frustrating to interact with the orthosis, computer and games. Despite their initial reservations, they decided to continue the therapy. Moreover, the positive results on IMI on group level indicate that participants perceived the training to be interesting and they enjoyed engagement with the system, which emphasizes the potential of this type of training for involving stroke patients actively in (prolonged) training.

Although a direct comparison between self-administered home training and a control group receiving comparable conventional treatment is lacking in the present study, the improvements in motor function of the arm in the present study correspond with those found in other robot-aided studies in chronic stroke in a clinical setting [14, 15, 17], and with therapy programs for the upper limb performed at home [10, 45, 46]. The improvements in our study involved particularly proximal arm movements. It is conceivable that reduction of arm weight did contribute to the gains presented by the proximal part of the FM, which is similar to results presented in previous studies [52, 53]. On individual level, eight participants (three mildly, four moderately, one severely impaired participant) achieved MCID of 10 % improvement for FM. Remarkably, seven of the 21 participants reached MCID of MAL, indicating that a third of the patients perceived a better use of the arm in daily activities, even though this was not reflected in an improved capacity as measured by ARAT. Of these seven patients, three were moderately and four mildly impaired. Hence, it seems that mild to moderate impaired patients benefit most from the intervention, which corresponds with previously reported robot-aided upper limb exercise training after stroke [54]. Furthermore, it is important to keep in mind that this study was conducted with chronic stroke patients. Ideally, home-based training should be considered at an earlier stage, for example as soon as inpatient rehabilitation is finished. This likely involves patients in the (sub)acute phase as well, where larger treatment effects would be expected. Although some participants did achieve clinically relevant gains in arm and hand function, others did not, underlining the need for further examination of which factors (for example age, time post stroke, stroke severity, training adherence, personal characteristics etc.) are associated with better treatment outcomes. This will assist in identifying who would benefit most from technology-supported, self-administered training at home, and ultimately, to find out for which patients this kind of home-based training is most suitable.

Many training studies using technology supported devices focus on either the proximal [14, 54] or distal [21, 55] arm only. Important aspects of the current study are that it aimed to involve functional movements of both the proximal and distal arm and hand within motivating rehabilitation games during training. The games available for practice mostly required movements of the arm, wrist and hand in sequence, like reaching, followed by wrist flexion or extension, and then hand opening and closing. This may have played a role in the limited effect on activity level, because proximal and distal movements may not have been integrated optimally. Although the games specifically incorporated hand gestures, these were rather coarse with generic flexion and extension of the thumb and fingers, and they did not contain specific functional grasps representing the handling of various objects. The expectation is that when such aspects are incorporated more specifically, exercises become even more functional and task-specific, which is likely to further enhance the clinical impact, predominantly on activity level [22, 56]. Therefore, integration of proximal and distal arm movements simultaneously, together with more functional and a larger variety of grasps, should be considered more specifically when designing games or exercises with a diversity of complexity for application in a training system dedicated to self-administered practice.

Although the arm function improvements in this study were comparable to those achieved through other robot-aided studies, they are still modest. An important factor might be the effective training duration. Although the average training duration was promising with approximately 105 minutes per week, especially in the light of effective use in other research into self-administered training [45, 50, 51], strong interindividual differences were observed. Qualitative information obtained during home visits revealed that some of the patients returned back to work or had a busy daytime schedule, limiting the time they had for the training. It is known that dose of robot-assisted training [18, 57] and conventional stroke rehabilitation [4, 58] is an important factor for clinical improvement, although an optimal or minimal dose is not yet known. The advised dose of 30 minutes per day, 6 days per week in this study (which totals to 18 hours in six weeks) corresponds closely with the 16 hours of additional training recommended by Kwakkel et al. to achieve clinically relevant functional improvements [59]. Observations on individual cases concerning a higher training duration and clinical improvements suggest that if an increase in training duration can be established, more pronounced clinical improvements might be achieved. Six participants who showed marked improvements in FM score over training had a rather high training duration (>100 minutes per week), which was also supported by the significant correlation between training duration and improvements in dexterity. In addition, the incorporation of a high variety of games probably enhances motivation during training, which might further stimulate a higher effective training duration. Additional motivational strategies might be implemented to increase participants’ effective training time during self-administered training even more. Future studies might consider approaches from the field of psychology to further explore this potential.

## Conclusions

In this study we evaluated the feasibility and potential clinical changes of self-administered and remotely monitored arm and hand training at home, with physical support from a dynamic wrist and hand orthosis and games representing exercises, in chronic stroke. Usability was perceived as sufficient and motivation was good, although issues were identified that need further improvements. Together with an effective use of 105 minutes per week, these findings indicate that home-based arm and hand training with physical support from a dynamic orthosis is a feasible tool to enable self-administered practice at home. Arm function improved, together with modest improvements in quality of life, indicating home-based arm and hand training can have clinical value, especially for mild to moderately impaired patients. By stressing the functional nature of the exercises even more in future applications, results on activity level may become more pronounced. Future research using a larger sample of participants including a control group should further examine ways to stimulate effective use and explore which factors are associated with better treatment outcomes, to identify those who would benefit most from this remotely supervised technology-supported training at home.

## Abbreviations

ADL: Activities of daily living
T01: Evaluation measurement one week before training
T08: Evaluation measurement within one week after training
T15: Evaluation measurement at two-month follow-up
HCP: Healthcare professional
SCRIPT: Supervised Care & Rehabilitation Involving Personal Telerobotics
SaeboMAS: Saebo Mobile Arm Support
IMI: Intrinsic motivation inventory
SUS: System usability scale
ARAT: Action research arm test
FM: Brunnstrom Fugl-Meyer assessment
MAL: Motor activity log
SIS: Stroke impact scale
SD: Standard deviation
IQR: Interquartile range
MCID: Minimal clinically important differences
